# Supplementary figures and images for: Genome-Wide Identification, Evolution, and Expression Characterization of the Pepper (Capsicum spp.) MADS-box Gene Family
Source: Genes (Basel). 2022 Nov 6;13(11):2047. doi: 10.3390/genes13112047 (PMC9690561; doi:10.3390/genes13112047)

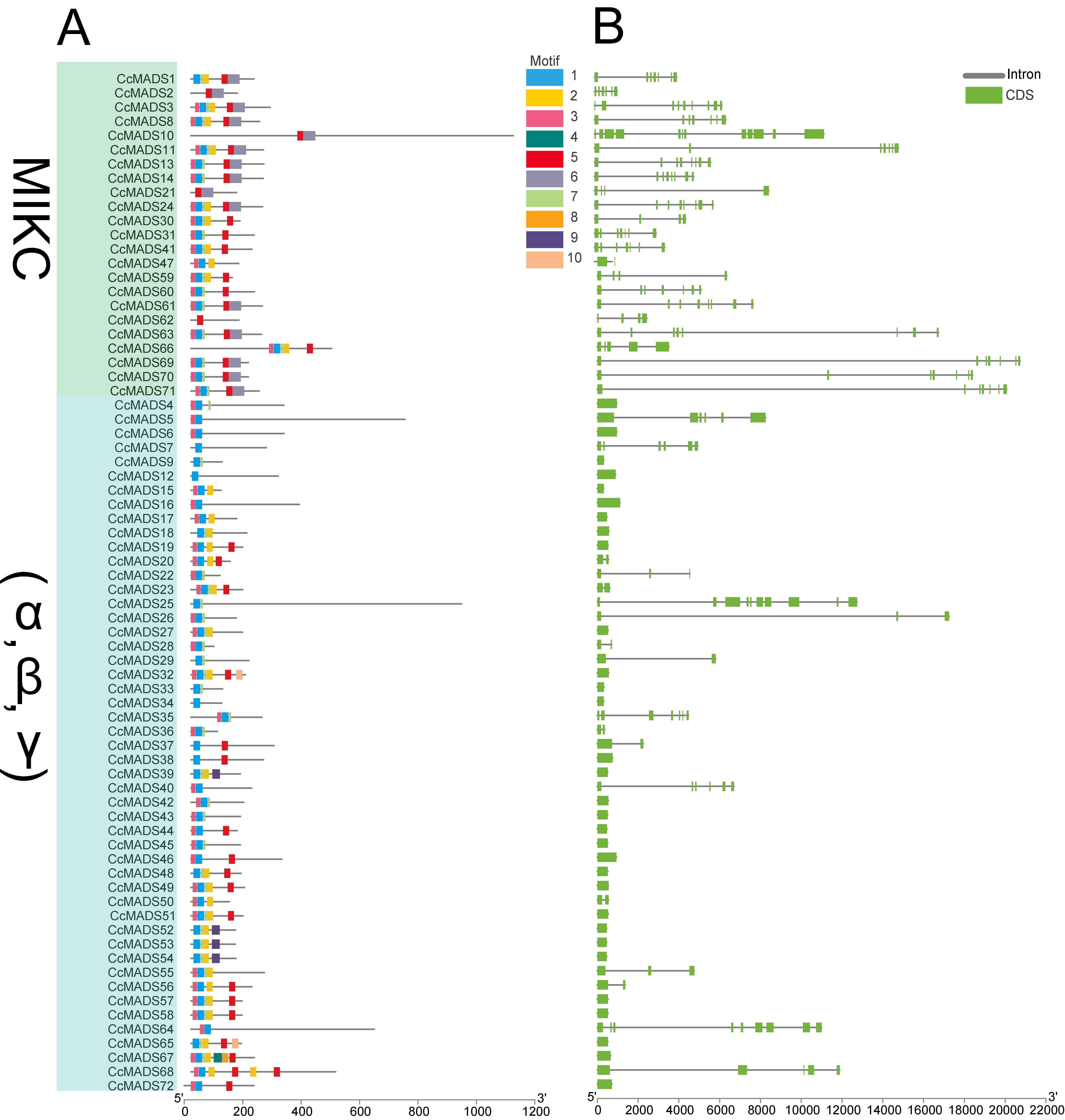

Supplement: Supplementary file 1 [file genes-13-02047-s001.zip › Figure S4.tif]

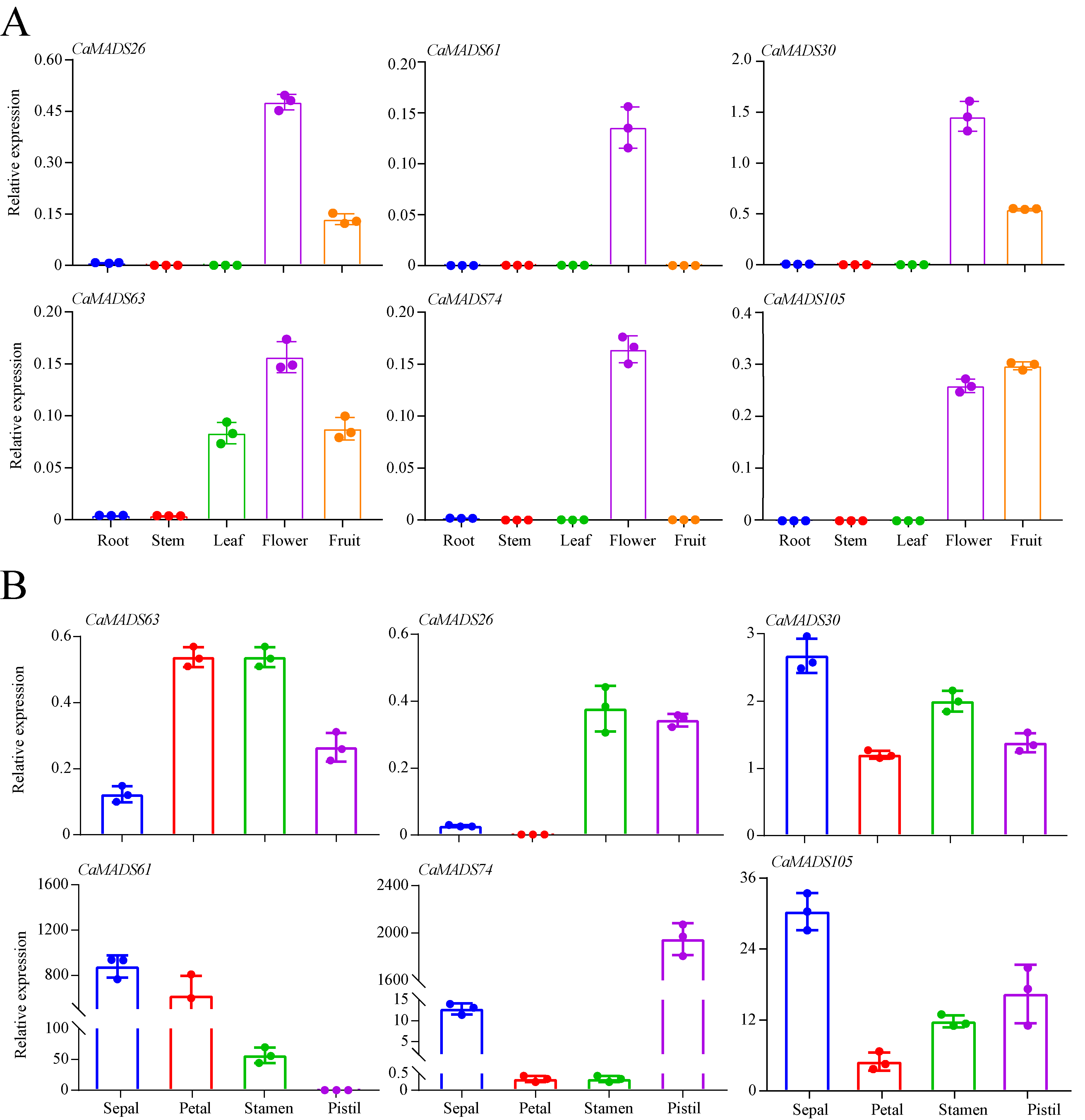

Supplement: Supplementary file 1 [file genes-13-02047-s001.zip › Figure S5.tif]
